# Participation of Single-Nucleotide Variants in *IFNAR1* and *IFNAR2* in the Immune Response against SARS-CoV-2 Infection: A Systematic Review

**DOI:** 10.3390/pathogens12111320

**Published:** 2023-11-06

**Authors:** María Fernanda López-Bielma, Ramcés Falfán-Valencia, Edgar Abarca-Rojano, Gloria Pérez-Rubio

**Affiliations:** 1 HLA Laboratory, Instituto Nacional de Enfermedades Respiratorias Ismael Cosío Villegas, Mexico City 14080, Mexicorfalfanv@iner.gob.mx (R.F.-V.); 2 Sección de Posgrado e Investigación, Escuela Superior de Medicina, Instituto Politécnico Nacional, Mexico City 11340, Mexico

**Keywords:** COVID-19, *IFNAR2*, antiviral response, *IFNAR1*, single-nucleotide variants

## Abstract

Host genetic factors significantly influence susceptibility to SARS-CoV-2 infection and COVID-19 severity. Among these genetic factors are single-nucleotide variants (SNVs). *IFNAR2* and *IFNAR1* genes have been associated with severe COVID-19 in populations from the United Kingdom, Africa, and Latin America. IFNAR1 and IFNAR2 are subunits forming the type I interferon receptor (IFNAR). SNVs in the *IFNAR* genes impact protein function, affecting antiviral response and disease phenotypes. This systematic review aimed to describe *IFNAR1* and *IFNAR2* variants associated with COVID-19 susceptibility and severity. Accordingly, the current review focused on *IFNAR1* and *IFNAR2* studies published between January 2021 and February 2023, utilizing the Preferred Reporting Items for Systematic Reviews and Meta-Analysis (PRISMA) protocol. The electronic search was conducted in PubMed databases using Boolean operators and inclusion and exclusion criteria. Of the 170 literature pieces, 11 studies were included. We include case reports of rare SNVs, defined by minor allele frequency (MAF) < 1%, and genome-wide associated studies (GWAS). Variants in *IFNAR1* and *IFNAR2* could potentially be new targets for therapies that limit the infection and the resulting inflammation by SARS-CoV-2 infection.

## 1. Introduction

**Figure 1:**
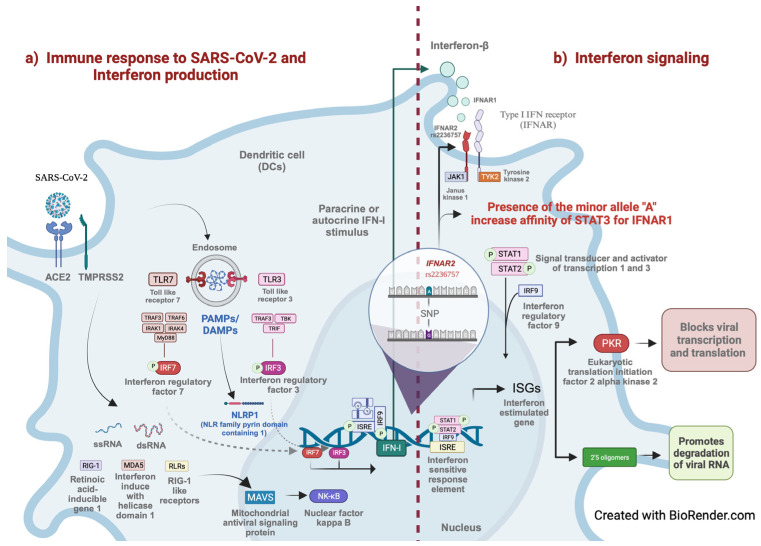
Entry of SARS-CoV-2 into the cell via ACE2 and TMPRSS2. The endosome is formed by double-membrane vesicles where the coronavirus replicates. Viral RNA (ssRNA, dsRNA) is recognized by TLR3, TLR7, RIG-I, and MDA5. These receptors activate TRIF and MyD88, downstream TLRs, and MAVS downstream RIG-I and MDA5, starting in the interferon production pathway. When IRF3, IRF7, or NFkB are activated, they translocate to the nucleus and trigger the transcription of immunogens like inflammatory cytokines and IFN. The dotted line represents the transition to IFN signaling, beginning with IFN-I binding IFNAR to initiate JAK/STAT signaling. In silico assays propose that the A allele of rs2236757 in *IFNAR2* increases the affinity of STAT3 to IFNAR1.

The interferon α/β receptor (IFNAR) comprises two subunits, IFNAR1 with a lower affinity for ligands than IFNAR2, which has a higher affinity. IFNAR2 is a type I membrane protein and is part of one of the two chains of a receptor for interferon (IFN), alpha and beta. IFNAR2 has three isoforms, the first of which is a non-functional protein with a truncated cytoplasmic domain. The second is lengthy and includes the functional trans-membrane protein together with IFNAR1. The soluble version of the receptor is the third form (sIFNAR2) [1]. The receptor activation stimulates STAT1 and STAT2 via Janus kinases (JAK2) [2]. After the activated STATs form, they translocate to the nucleus. Subsequently, it initiates transcription by binding specific sites in IFN-stimulated gene (ISG) promoters. A critical transcriptional complex induced by type I IFNs is the ISG factor 3 (ISGF3) complex. The mature ISGF3 complex comprises the phosphorylated (activated) forms of STAT1, STAT2, and IRF9. The transcription initiation is by the complex binds specific IFN-stimulated response elements (ISREs) present in the promoters of certain ISGs (Figure 1). Other STAT complexes induced by type I IFNs include homodimers (STAT1, STAT3 to STAT6) or heterodimers STAT1 with STAT2 to STAT6. Such IFN-induced complexes bind to the IFN-γ-activated (GAS) element in the ISGs’ promoter. Some known ISGs have ISREs, GAS, or both elements in their promoters [3,4].

Several proteins known as IFN-stimulated gene products with antiviral, antiproliferative, and immunomodulatory functions are secreted due to the interaction between IFN type I and IFNAR [5]. The RNA-activated protein kinase is one of these products (PKR). Viral dsRNA mediates the activation of PKR; when a virus activates PKR, it phosphorylates the subunit of initiation factor 2 (IF2), which reduces mRNA translation and prevents the creation of viral proteins [6]. IFN triggers the activation of 2′–5′ oligoadenylate (2′–5′A) production. The 2′–5 activates RNase, which causes viral degradation [5].

Genetic variants in *IFNAR1* and *IFNAR2* could affect the signaling pathway differently, such as decreased protein abundance and impaired internalization in response to interferon or its interaction. An example of this is the homozygosity for a nonsense *IFNAR2*. This variant was first reported in two siblings. It became known when the proband received the live-attenuated measles, mumps, and rubella (MMR) immunization and developed fatal encephalitis exacerbated with hemophagocytic lymphohistiocytosis [6]. Single-nucleotide variants (SNVs) are the most frequent among other genetic variants. The presence of rs201609461 allele T in *IFNAR1* encodes a truncated protein absent from the cell surface [7]. The A allele in rs1601861199 creates a premature stop codon, while the G allele in rs1601861196 originates a modified splice site for *IFNAR1* [8]. On the other hand, variants in *IFNAR2* have been associated with a risk of persistent hepatitis B virus infection (rs2229207 and rs1405333677) [9] or code *IFNAR2* truncate (rs1310889473 and rs1312285586), causing dysregulation of NK cell functions [10].

Various viruses called coronaviruses can lead to mild and severe respiratory infections in humans. Among these, two zoonotic coronaviruses are the Middle East respiratory syndrome coronavirus (MERS-CoV) and the severe acute respiratory syndrome coronavirus (SARS-CoV). Another new coronavirus, SARS-CoV-2, caused a distinct viral pneumonia in Wuhan, China, at the end of 2019 [11].

In the airway, SARS-CoV-2 moves down, reaching alveolar epithelial cells in the lungs and replicating after attaching to respiratory epithelial cells [11]. SARS-CoV-2 uses the same receptor as SARS-CoV, angiotensin-converting enzyme 2 (ACE2), as the entry receptor and employs the cellular serine protease TMPRSS2 for S protein priming [12]. RNA viruses such as SARS-CoV-2 are detected in the endosome by dendritic cells (DCs) through Toll-like receptor 7 (TLR7), while cytosolic RNA sensors such as RIG-I, MDA-5, and RIG-I-like receptors (RLRs) can detect double-stranded RNA (dsRNA) during viral propagation. Downstream signaling stimulates the transcription of IRF3/IRF7-dependent type I and type III IFNs in response to interaction with TLR and RLRs [13]. Upon infection with respiratory SARS-CoV-2, signaling cascades are activated, producing IFN, which activates IFN signaling pathways both autocrinally and paracrinally. The primary producers of IFN during respiratory virus infection are epithelial cells, alveolar macrophages (AMs), natural killer (NK) cells, DCs, and immune memory macrophages (IMMs) [14]. DCs produce high levels of IFNs and are likely the main contributors to IFN expression in response to coronavirus infection [5]. SARS-CoV-2 proteins interfere with the signaling pathways of IRF3 interference, and ORF6, ORF8, and nucleocapsid proteins of SARS-CoV-2 repress type I interferon (IFN-β) activation. The S protein of SARS-CoV-2 stimulates the production of the suppressor of cytokine signaling (SOCS3/1) in infected cells as an additional viral tactic. The SOCS3/1 inhibits IFN synthesis and boosts SARS-CoV-2 replication in infected cells [15].

SARS-CoV-2 infection causes the disease COVID-19. The symptoms of COVID-19 range from entirely asymptomatic to severe illness and death, but the most common symptoms include fever, fatigue, and dry cough. Less common symptoms are diarrhea, anorexia, nausea, and vomiting. Pneumonia, acute respiratory distress syndrome, liver damage, heart damage, thrombosis, renal disease, neurologic disease, and sepsis are just a few of the complications that can cause severe sickness and even death [16].

Currently, there is no curative treatment for COVID-19; however, some strategies have shown benefits in specific populations. In the early phase of COVID-19, the triple combination of interferon beta-1b, lopinavir/ritonavir, and ribavirin therapy was used in a group of mild to moderate COVID-19 patients from Hong Kong and China. Triple therapy has been demonstrated to reduce symptoms, speed up virus shedding, and reduce hospital stays [17].

In COVID-19, older age, male sex, and comorbidities affect the clinical course of disease and outcome. Nevertheless, these aspects are merely responsible for a portion of the differences in severity between individuals. Genetic variations across populations are linked to illness susceptibility or immune response outcomes. The most frequent genetic variations are single-nucleotide variants (SNV). These SNVs can occur in coding or non-coding areas of genes, leading to function loss or alteration, modifying amino acid sequences and protein structure, or using alternative splicing methods [18,19]. An approach to identify SNVs associated with the disease is genome-wide association study (GWAS) and candidate gene study [20]. These assays have been integral in identifying SNVs associated with COVID-19 severity and susceptibility to SARS-CoV-2 infection. A GWAS conducted on a population from Spain and Italy with severe COVID-19 uncovered six potential genes with COVID-19-related activities [21]. For example, SNV rs657152 (A allele) in the ABO group associated with COVID-19 showed that blood group A has higher susceptibility and respiratory failure rate than other blood groups; the mechanism of how blood group might affect susceptibility remains to be clarified [21].

This systematic review aimed to describe SNV in *IFNAR1* and *IFNAR2* genes associated with a high risk of viral infection or clinical relevance in COVID-19. To do this, we pose the following research question: do the risk variants in SNVs of *IFNAR1* or *IFNAR2* of patients with severe COVID-19 affect the antiviral immune response or lead to poor outcomes compared to noncarriers individuals of risk variants?

There are two approaches for finding genes and genetic variants involved in common diseases: linkage studies and genetic association studies. In complex diseases, one particular genetic variant is not sufficient or necessary for disease development. There is also the influence of interactions between other genes, environment, epigenetics mechanisms, and microbiome; however, identifying genetic variants associated with disease contributes to the best understanding of the disease.

## 2. Materials and Methods

### 2.1. Search Strategies

Following the PRISMA guidelines [22], a comprehensive literature search was performed to identify relevant studies on “The association of *IFNAR1* and *IFNAR2* variants with COVID-19 severity or mortality in different populations”. The search aimed to identify studies published in English between January 2021 and February 2023.

A search strategy was conducted reviewing in PubMed using the Medical Subject Headings (MeSH) terms. The primary search terms included “IFNAR”, “IFNAR1”, “IFNAR2”, “polymorphism”, “GWAS”, “viral infection”, COVID-19”, “Influenza”, and “receptor, interferon alpha-beta”. We used the following syntax constructed (“Receptor, interferon alpha-beta” [MeSH] AND “Polymorphism, Genetic” [MeSH]), (IFNAR protein, human” [Supplementary Concept]) AND “Polymorphism, Genetic [Mesh], (“IFNAR2 protein, human” [Supplementary Concept]) AND “COVID-19” [Mesh], (“IFNAR2 protein, human” [Supplementary Concept]) AND “ Influenza, human/genetics” [Mesh], (“IFNAR2 protein, human” [Supplementary Concept]) AND “Influenza, Human” [Mesh], (“IFNAR2 protein, human” [Supplementary Concept]) AND “Polymorphism, Genetic” [Mesh], (“IFNAR2 protein, human” [Supplementary Concept]) AND “Genome-Wide Association Study” [Mesh], ((“IFNAR2 protein, human” [Supplementary Concept]) AND “Polymorphism, Single Nucleotide [Mesh]) AND “COVID-19” [Mesh], (“IFNAR protein, human” [Supplementary Concept]) AND “Polymorphism, Genetic” [Mesh], ((“IFNAR protein, human” [Supplementary Concept]) AND “Polymorphism, Genetic” [Mesh]) AND “Virus Diseases” [Mesh], ((“IFNAR2 protein, human” [Supplementary Concept]) AND “Virus Diseases” [Mesh]) AND “Polymorphism, Single Nucleotide” [Mesh], ((IFNAR2) AND (VIRAL RESPONSE)) AND (POLYMORPHISM), and (“Receptor, Interferon alpha-beta” [Mesh]) AND “Polymorphism, Genetic” [Mesh].

### 2.2. Inclusion Criteria

We included gene candidate association studies and genome-wide association studies (GWAS) from January 2021 to February 2023. Articles were included based on the PICO criteria: (P) Population: people with COVID-19. (I) Intervention: evaluating the association of single-nucleotide variants (SNVs) in *IFNAR1* or *IFNAR2* in viral infection or disease progression. (C) Comparison: compared with people without viral infection, asymptomatic or mild to moderate COVID-19. (O) Outcome: SNV associated with the affectation of an antiviral immune response or poor disease outcome (Table 1).

### 2.3. Data Collection

We used a multiple-assessment technique to find, filter, and select studies according to the PRISMA standards [22]. Two investigators (MFLB and GPR) performed the literature review independently. The research team met frequently to discuss the project while iterating through screening, analysis, and synthesis. The Guide for Evaluation of Qualitative Research Studies (GEQRS) was used to evaluate the literature [23].

**Figure 2:**
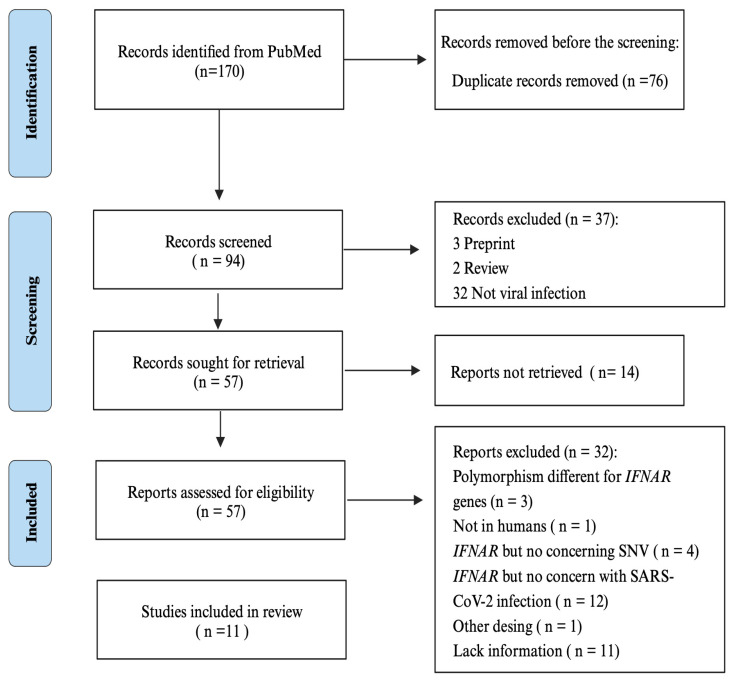
Flow diagram for the included studies.

After duplicated removal, one reviewer applied the inclusion and exclusion criteria by screening the title and abstract and examining the full text. MFLB extracted information using the guidance on conducting systematic scoping reviews [24]. At the same time, GPR crosschecked for completeness. RFV and EAR utilized the quality appraisal methodology to ensure the reliability and validity of the research [22,25]. Because of the potential for bias, articles with evaluation scores lower than 70% were excluded (Figure 2).

## 3. Results

Recently, an increasing number of investigations have focused on studying host genetic factors contributing to severe COVID-19. Using GWAS, *IFNAR2* variants were related to susceptibility, COVID-19 severity, or poor disease outcome.

GWAS in critical patients with COVID-19 recruited from GenOMICC (Genetics Of Mortality In Critical Care) from 208 intensive care units in the United Kingdom showed that A allele of rs2236757 in *IFNAR2* was associated (*p* = 4.9 × 10^−8^, OR = 1.28) with needing mechanical ventilation, supplementary oxygen, or having pneumonia associated to COVID-19. Transcriptome-wide Mendelian randomization suggested that critical COVID-19 was related to interferon pathways [26]. GWAS of 52,630 patients of four cohorts with COVID-19 replicated the association with disease severity and rs2236757 of *IFNAR2* [27]. In patients with severe COVID-19 and non-critical patients from Brazil, the rs2236757 did not show a significant difference in white people; however, in non-white patients, the AA genotype, by recessive model, was associated (*p* = 0.045) with worse clinical outcomes (OR = 3.23) like admission to the intensive care unit. Furthermore, other genes such as *TMPRSS2* and *ACE1* were associated with the mortality of COVID-19 patients (OR = 6.468, 95% CI 1.463–28.592, *p* = 0.014) (Table 2) [28].

GWAS in the European population with severe COVID-19 showed that the C allele of rs2834161 (*IFNAR2*) was associated with respiratory failure (*p* = 6.1 × 10^−7^, OR = 1.25). Both rs2834161 and rs12610495 (*DPP9)* showed strong but no statistically significant tendencies for association with severe COVID-19 in younger people (Table 2) [29]. Notably, rs2236757 was the most replicated in the Caucasian population; this variant did originate from Mendelian randomization (MR) analysis and showed evidence that lower expression of *IFNAR2* is associated with life-threatening COVID-19 [26]. The summary databased Mendelian randomization (SMR) method identified rs2252639 of *IFNAR2* associated (*p* < 1 × 10^−4^) with severe COVID-19 and an association with COVID-19 hospitalization (*p* < 1 × 10^−6^); the authors assume that *IFNAR2* is involved in determining COVID-19 severity and could be a potential therapeutic target for the treatment of COVID-19 [30]. On the other hand, Mendelian randomization showed significant results for the variant rs13050728 (*IFNAR2*), with the strongest correlation with COVID-19 hospitalization (OR = 1.17, *p* = 1.88 × 10^−12^). This variant is in the intron; however, MR shows that rs13050728 is strongly correlated (r^2^ > 0.75) with other SNVs of *IFNAR2* (rs11911133, rs1051393, rs2300370, rs56079299, rs17860115, rs2236758, rs12053666, and rs1131668) [31].

With a meta-analytic method using results of GWAS in the Caucasian population, a novel variant in the *IFNAR2-IL10RB* region (rs9976829, G allele) was associated with a 16% greater chance of COVID-19 infection in comparison to carrying patients of A allele [32].

In a validation study in the Mexican population, the association (*p* = 0.03) of rs2236757 (AG or GG genotype) with mortality risk (OR = 1.43) in severe COVID-19 patients was replicated. rs2834158 and rs3153 were also evaluated, identifying by the dominant model that patients’ carriers of TC or CC (rs2834158) and carriers of AG or GG (rs3153) showed significative association with mortality risk (*p* = 0.027, OR = 1.38 and *p* = 0.019, OR = 1.39, respectively); this is one of the first reports where they indicate association (*p* = 0.040) at the haplotype level (rs3153/rs2229207/rs1051393/rs2834158, GTGC) and mortality risk (OR = 2.08) among patients with severe COVID-19 [33].

A Vietnamese study reported the variants rs17860118 and rs2229207 in *IFNAR2* associated with SARS-CoV-2 susceptibility (*p* = 0.033, OR = 1.718 and *p* = 0.012, OR = 1.89, respectively) but no statistically significant association with severe COVID-19. It should be noted that the rs2229207 (T > C) is in the exonic region, and the presence of risk allele (C) at the level of protein codified to serine instead of phenylalanine [34]. This change induces an increased response to IFNα and β [35] (Table 2).

**Table 2:** SNV in *IFNAR2* and other genes associated with COVID-19.

Gene	SNV	RA/RG	MAF * (%)	Loci	Gene Consequence	OR	*p*-Value	Population	Associated Outcome	Reference
*IFNAR2*	rs2236757	A	0.34_gcc_ 0.28_ukb_	21q22.1	Intront variant	1.28	4.9 × 10^−8^	United Kingdom		[26]
			0.29			1.28	7.0 × 10^−5^	Europe, Africa, South Asia, and Latin America	Severity Mortality	[27]
			0.42			3.23	0.045	Brazil, non-white		[28]
*TMPRSS2*	rs12329760	T	0.27	21q22.3	Missense variant	NR	0.04	Europe, Africa, South Asia, and Latin America	Severity	[28]
*ACE1*	rs1799752	Ins	0.41	17q23.2	Intront variant	NR	0.01			
*IFNAR2*	rs2834161	C	0.37	21q22.1	Intront variant	1.25	6.10 × 10^−7^	Italy, Spain, Norway, Germany, and Australia	Mortality	[29]
	rs13050728	T	0.40	21q22.1	Intront variant	1.17	6.10 × 10^−12^	Caucasian	Severity	[31]
	rs9976829	G	0.33	21q22.1	Intront variant	1.16	2.57 × 10^−6^	Caucasian	Susceptibility	[32]
	rs2834158	TC, CC	0.46 0.18	21q22.1	Intront variant	1.38	0.027	Mexican	Mortality	[33]
	rs3153	AG, GG	0.46 0.19	21q22.1	Intront variant	1.39	0.019			
	rs17860118	T	0.18	21q22.1	5’ UTR	1.72	0.033	Vietnamese	Susceptibility	[34]
	rs2229207	C	0.19	21q22.1	Missense variant	1.89	0.012			

SNV, single-nucleotide variant; RA, risk allele; RG, risk genotype; * MAF, minor allele frequency reported in each study; gcc, GenOMICC European cohort; ukb, UK Biobank; NR; non-reported; ins, insertion; UTR, untranslated region.

Through GWAS, genetic variants in *IFNAR2* located in introns and risk of severe COVID-19 or with poor prognosis have been associated and replicated; however, there are few studies of association with SNVs in exons or promoter regions; it should be noted that associated variants in COVID-19 had low frequency in the population.

Whole-genome sequencing (WGS) in a multiethnic cohort from the ODYSSEY (Once-daily DTG-based ART in Young people vs. Standard thErapY) showed that A allele (rs72550721) was associated with severe COVID-19. Among other interesting findings from this study were different rare variants (MAF < 1%) of *IFNAR2* detected among hospitalized severe COVID-19-positive patients (Table 3). They report that populations of Asian descent had more frequent variants than the rest of the populations analyzed [36].

There are few genetic association studies of *IFNAR1* in COVID-19 in the literature. Compared to *IFNAR2*, studies of genetic variants of *IFNAR1* and COVID-19 had a small sample size; however, the results in *IFNAR1* and infection by SARS-CoV-2 are relevant. Patients with *IFNAR1* deficiency develop a severe form of COVID-19. The rs1601861196 (A > G) is in a splice site, and the GG carriers show IFNAR1 deficiency. This novel variant reveals the importance of the anti-viral role of type I IFN and should be considered in the treatment of these patients with IFN administration; unfortunately, it is unknown the frequency of risk alleles in the population [37].

Next-generation sequencing identified two SNVs in *IFNAR1* (rs181939581, G/C) and rs746291558, A/C). These polymorphisms cause a change in the amino acid sequence (Trp73Cys and Ser422Arg, respectively); serum of patients’ carriers of risk allele and with severe COVID-19 show low levels (<1 pg/mL) of IFN-α [38].

## 4. Discussion

The IFN signaling pathway shows the relevance of this mechanism in regulating immune response during viral infections; however, viruses have mechanisms of IFN pathway evasion, and SARS-CoV-2 is no exception [39]. In addition to the already-known risk factors for COVID-19, genetic variants contribute to the severe disease [40].

There are even findings that indicate that population ancestry contributes to the occurrence of severe COVID-19. An example is rs2236757, which is the SNV more associated and replicated to severe COVID-19; the minor allele (A) of rs2236757 has presented recent positive selection only in the African population. The linkage disequilibrium (LD) analysis showed that rs2236757 and rs2073361 have higher LD (r^2^ = 1) in comparison with other populations [41]. According to in silico analysis, this variant in *IFNAR2* can increase the affinity of STAT3 for elements connected to *IFNAR1, PAXBP1, GART, C21ORF49, SON*, and *IL10RB* [42]. The rs2236757 variant interacts with *TMPRSS2* and *ACE1*, influencing COVID-19 severity and mortality. For example, in non-white patients, the minor alleles of rs2236757 (*IFNAR2*) and rs12329760 (*TMPRSS2*) increase, in an additive way, the risk of death [28].

On the other hand, the presence of the mutation in *IFNAR1* (Exon6:c.674(-2b)A  >  G) causes a deficiency of the corresponding protein. In these patients, there is a probability that, after the administration of interferon, they will not have a favorable result [37]. Rare variants in *IFNAR2* (rs72550721, A) produce a Tyr322Ter change that results in a stop codon, causing a loss of function in *IFNAR2*, which in the Asiatic population was associated with increased susceptibility to severe COVID-19 [36]. IFNAR2 is important in COVID-19, as evidenced by type-I IFN gene expression decreasing following SARS-CoV-2 infection. Patients who express IFNAR2 poorly are more likely to be hospitalized for COVID-19 [31]. Currently, there are genetic variants that have been associated with increased expression of *IFNAR2* (T allele, rs9975538); the authors concluded that this variant might be upregulating the antiviral activity and conferring protection by increasing IFN receptor [43]. Another risk variant associated with increased *IFNAR2* expression in classical monocytes was rs13050728 (allele T); in the Japanese population, the risk allele might contribute to severe COVID-19; these patients showed a low fraction of non-classical monocytes [44].

Through molecular docking analysis, the rs768348126 variant showed decreased hydrogen bonds formed between the IFNAR2 protein and IFN-α. With the common allele (C), 13 bonds were formed, while with the risk allele (G), only 6 were created, which destabilizes the binding with the IFN-α, a molecule in response to COVID-19 [45].

Rare variants in the population are also of relevance; rs1987287426 (*IFNAR2*; T > C) is a coding sequence variant (serine > proline). The presence of proline prevented cell-surface expression of IFNAR2 and persisted intracellularly in an aberrantly glycosylated state. In vitro assay of cells with risk alleles showed vulnerability to multiple viruses [46].

The relevance of studying genetic variants in *IFNAR1* and *IFNAR2* in patients with COVID-19 is that there are previous reports of polymorphisms in these genes associated with response to interferon therapy, like rs9984273 (*IFNAR2*) located in a binding motif for the glucocorticoid receptor. Patients with acute respiratory distress syndrome (ARDS) carriers of the C allele (rs9984273) showed decreased mortality in the IFN β treatment arm compared to patients with the T allele [40].

### 4.1. Participation of SNVs in IFNAR1 and IFNAR2 in Other Viral Infections

The variants associated with mortality risk in COVID-19 Mexican patients (rs2236757, rs2834158, rs3153, and rs1051393) [33] were previously explored in European American and African American patients from the study HALT-C (Hepatitis C Antiviral Long-Term Treatment Against Cirrhosis). rs2834158, rs3153, and rs2236757 (*IFNAR2*) have not been significantly statistically associated with treatment response in chronic hepatitis C patients. However, other genetic variants of *IFNAR2* with interesting associations in viral disease have been reported [35].

Chronic hepatitis C patients’ carriers of the rs2229207 C allele (IFNAR2) and combined treatment with pegylated-interferon-alpha (PEG IFN-alpha) and ribavirin showed an association (*p* = 0.02) with the increased risk (OR, 2.07) of presenting a sustained virological response (SVR). Additionally, patients’ carriers of the GT or GG genotype (rs2243592/*IFNAR1*) showed lower frequency in patients with SVR; on the contrary, patients with genotype TC or CC (rs4986956/*IFNAR2*) were highly frequent in SVR [35]. At amino acid position 8, the rs2229207 (*IFNAR2*) mutation converts phenylalanine to serine [35]. On the other hand, the rs2229207 has been reported as a risk for persistent hepatitis B virus infection; in an in vitro assay, the aminoacid change alters IFNAR2 protein trafficking to the membrane or secretion of the truncated forms of the protein [9].

Another variant of interest was rs1051393; carriers of the T allele show a risk of chronic Hepatitis B virus infection (*p* = 0.077, OR = 1.24). rs1051393-T is a missense SNP altering amino acid from phenylalanine to valine. This SNP is located on the signal peptide region, crucial in IFNAR2 protein trafficking to the membrane. rs1051393T might be important in the risk of HBV infection by affecting IFNAR2 protein expression on the cell surface, leading to antiviral response and impaired signal transduction; however, is necessary to conduct further experiments to test this hypothesis [47].

In hepatitis viral infected patients from Wuhan, China, the intron variant rs1012335 (C allele) and missense variant rs2257167 (C allele) are associated with acute and chronic hepatitis B liver failure and hepatocellular carcinoma, showing that the variants can not only participate in early-stage pathogenesis of hepatitis B viral infection. A strongly linked disequilibrium was also found within these two alleles (*p* < 0.001) in the study population [48]. According to the treatment response in the Japanese population, rs2243594 (G allele, *IFNAR1*) was associated with neutropenia in chronic hepatitis C patients receiving interferon (IFN) plus ribavirin combination therapy. This is very important to consider because the therapy with ribavirin may present hematologic toxicities [49].

The role of the IFN pathway as an anti-viral agent has been well documented for a long time; however, in COVID-19, additional research is necessary. We should consider, in addition to the already-known risk factors (sex, comorbidities, and age), the genetic ancestral contribution and SNV in IFN receptors.

### 4.2. Perspectives

High-risk groups that could benefit from IFN-α/IFN-β therapy could be identified by recognizing patients with low IFN production but preserved cellular responsiveness to type I IFN [50]. Recombinant and pegylated IFN-α and IFN-β are currently indicated in hepatitis B or C and other non-infectious diseases such as multiple sclerosis. IFN-λ was discovered to be more successful than IFN-α in preventing and treating influenza virus infection in mice, with no increase in inflammation or tissue damage when compared with IFN-α [51]. However, because IFNAR is expressed widely, the IFN-α/IFN-β has severe systemic adverse effects [52]. The IFNAR1 absence affects signaling measure by 2′-5′A, antiviral and antiproliferative. On the other hand, IFNAR2 absence appears unable to traduce signals as measured in this study [53]. IFNs might facilitate improved treatment of viral infection with high anti-viral and T-helper 1-inducing activity but low antiproliferative activity, which might cause dosing-limiting effects such as neutropenia or thrombopenia [54].

In vitro, SARS-CoV-2 replication is inhibited by IFN-α and IFN-β [55]. Interestingly, SARS-CoV-2 has a much higher sensitivity to type I IFN than other coronavirus. The authors proposed that this augmented type I IFN sensitivity is likely due to genetic differences in viral proteins between viral strains [56]. Using a cell line model, IFN-β strongly upregulated the expression of canonical antiviral ISGs and ACE2 at the mRNA and cell-surface protein levels. In vitro assays showed that it inhibited SARS-CoV-2 replication in a dose-dependent manner and showed potent antiviral activity [57]. In models of primary human airway epithelial (pHAE) cultures infected with SARS-CoV-2, type I and III IFNs reduced virus replication and correlated with upregulation of antiviral effector genes with a predominance of pro-inflammatory cytokines and chemokine induction (IL-6, TNF-α, and CXCL8). With this evidence, the authors proposed that both type I and III IFNs can be therapeutic options for treating COVID-19 patients [58].

In a multicenter, prospective, open-label, randomized, phase 2 trial in adults with COVID-19 in six hospitals in Hong Kong, the efficacy and safety of combined interferon beta-1b, lopinavir-ritonavir, and ribavirin were assessed. This therapy was safe and superior to ribavirin, enhancing virus shedding, alleviating symptoms, and reducing the days of hospital stay of patients with mild to moderate COVID-19 [17]. Patients with severe COVID-19 with IFNAR1 deficiency treated with IFN-γ showed increased oxygen saturation (SpO2) to 94% in the nine days of hospitalization [37].

The acute inflammation seen in COVID-19 patients results from repressed type I IFN expression and an imbalance in IFN response, which may be corrected with IFN therapy.

The first days of infection should be considered in therapeutic type I IFN [59]; however, further investigation of patients with multiple comorbidities or who were diagnosed late COVID-19 [58]. It is necessary to consider the genetic variation in receptors of IFN or patients with IFNAR1 deficiency [35]. Studies have demonstrated that IFNAR is rapidly downregulated by chronically administered type I IFN therapy. On the contrary, IFNAR-1, which is not modulated during IFN-β therapy, was correlated to poor response to IFN-β therapy as a cause of hyperactivity of the type I IFN pathway [60].

We consider genomic variations in *IFNAR1* and *IFNAR2* genes relevant to the IFN-therapy responsiveness, for or against. Previously, the importance of IFN binding to IFNAR1 in proinflammatory responses has been characterized. This study discovered a significant contact interface that is mediated by two IFNAR1 residues (Tyr^240^ and Tyr^274^) interacting with IFN- β residues (Phe^63^, Leu^64^, Glu^77^, Thr^78^, Val^81^, and Arg^82^). The identified interaction is important as it governs all aspects of IFN-β functionality, stabilizes the ligand–receptor complex, and could serve as a viable target for drug design. In the murine model, IFN-β and IFN-γ partially utilized these residues for an efficient ISRE-dependent response. The interface spanning these two residues may be a site of species or IFN subtype specificity. Therefore, a variant in this domain may affect the interaction interface and the treatment response [61].

Synthetic IFN receptors might help unravel general IFNAR signaling or help analyze the biological consequences of patient-derived non-synonymous single-nucleotide variants in IFNARs [62].

This systematic review highlights the need to expand population genetic studies to identify new variants associated with mortality or susceptibility to COVID-19 and their frequencies in the risk population. SNVs of clinical relevance in viral infections such as hepatitis B or C should be explored in COVID-19.

## 5. Conclusions

Single-nucleotide variants in the *IFNAR1* and *IFNAR2* genes potentially affect the immune response, exacerbate, or attenuate the disease in the face of SARS-CoV-2 infection, in addition, could be new targets for therapies that limit the infection and the resulting inflammation.

## Figures and Tables

**Table 1:** Search strategy criteria.

**Inclusion criteria**	
Population	Patients with COVID-19.
Outcome	SNV in *IFNAR1* and *IFNAR2* is associated with the affectation of an antiviral immune response or poor disease outcome.
Types of study design	Observational, case-control, cross-sectional, case reports, and genome-wide association studies (GWAS).
**Exclusion criteria**	
Studies on patients who had infections viral infections different from COVID-19.	
Studies with incomplete or lacking necessary data.	
Studies in pregnant women.	
Duplicate studies.	
Studies not in the English language.	

**Table 3:** Rare variants (MAF < 1%) of *IFNAR2* associated with severe COVID-19.

SNV	Gene Consequence	Minor Allele	MAF (%), 1000 Genomes
rs72550721	Stop gained	A	0.70
rs144384060	Missense variant	A	0.12
rs749487628	Missense variant	T	0.00 *

* Exome Aggregation Consortium.

## Data Availability

Data sharing not applicable.

## References

[1] Órpez-Zafra T., Pavía J., Hurtado-Guerrero I., Pinto-Medel M.J., Bada J.L.R., Urbaneja P., Suardíaz M., Villar L.M., Comabella M., Montalban X. (2017). Decreased soluble IFN-β receptor (sIFNAR2) in multiple sclerosis patients: A potential serum diagnostic biomarker. Mult. Scler. J..

[2] Bastard P., Hsiao K.-C., Zhang Q., Choin J., Best E., Chen J., Gervais A., Bizien L., Materna M., Harmant C. (2022). A loss-of-function *IFNAR1* allele in Polynesia underlies severe viral diseases in homozygotes. J. Exp. Med..

[3] Murphy K., Travers P., Walport M. (2008). Janeway’s Immunobiology.

[4] Platanias L.C. (2005). Mechanisms of type-I- and type-II-interferon-mediated signalling. Nat. Rev. Immunol..

[5] Sodeifian F., Nikfarjam M., Kian N., Mohamed K., Rezaei N. (2022). The role of type I interferon in the treatment of COVID-19. J. Med Virol..

[6] Duncan C.J., Randall R.E., Hambleton S. (2021). Genetic Lesions of Type I Interferon Signalling in Human Antiviral Immunity. Trends Genet..

[7] Zhang Q., Bastard P., Bolze A., Jouanguy E., Zhang S.-Y., Cobat A., Notarangelo L.D., Su H.C., Abel L., Casanova J.-L. (2020). Life-Threatening COVID-19: Defective Interferons Unleash Excessive Inflammation. Med.

[8] Hernandez N., Bucciol G., Moens L., Le Pen J., Shahrooei M., Goudouris E., Shirkani A., Changi-Ashtiani M., Rokni-Zadeh H., Sayar E.H. (2019). Inherited IFNAR1 deficiency in otherwise healthy patients with adverse reaction to measles and yellow fever live vaccines. J. Exp. Med..

[9] Frodsham A.J., Zhang L., Dumpis U., Taib N.A.M., Best S., Durham A., Hennig B.J.W., Hellier S., Knapp S., Wright M. (2006). Class II cytokine receptor gene cluster is a major locus for hepatitis B persistence. Proc. Natl. Acad. Sci. USA.

[10] Passarelli C., Civino A., Rossi M.N., Cifaldi L., Lanari V., Moneta G.M., Caiello I., Bracaglia C., Montinaro R., Novelli A. (2020). IFNAR2 Deficiency Causing Dysregulation of NK Cell Functions and Presenting With Hemophagocytic Lymphohistiocytosis. Front. Genet..

[11] Hu B., Guo H., Zhou P., Shi Z.-L. (2021). Characteristics of SARS-CoV-2 and COVID-19. Nat. Rev. Microbiol..

[12] Hoffmann M., Kleine-Weber H., Schroeder S., Krüger N., Herrler T., Erichsen S., Schiergens T.S., Herrler G., Wu N.-H., Nitsche A. (2020). SARS-CoV-2 Cell Entry Depends on ACE2 and TMPRSS2 and Is Blocked by a Clinically Proven Protease Inhibitor. Cell.

[13] A Madden E., Diamond M.S. (2022). Host cell-intrinsic innate immune recognition of SARS-CoV-2. Curr. Opin. Virol..

[14] Lowery S.A., Sariol A., Perlman S. (2021). Innate immune and inflammatory responses to SARS-CoV-2: Implications for COVID-19. Cell Host Microbe.

[15] Ahmed C.M., Grams T.R., Bloom D.C., Johnson H.M., Lewin A.S. (2022). Individual and Synergistic Anti-Coronavirus Activities of SOCS1/3 Antagonist and Interferon α1 Peptides. Front. Immunol..

[16] Merad M., Blish C.A., Sallusto F., Iwasaki A. (2022). The Immunology and Immunopathology of COVID-19. Science.

[17] Hung I.F.-N., Lung K.-C., Tso E.Y.-K., Liu R., Chung T.W.-H., Chu M.-Y., Ng Y.-Y., Lo J., Chan J., Tam A.R. (2020). Triple combination of interferon beta-1b, lopinavir–ritonavir, and ribavirin in the treatment of patients admitted to hospital with COVID-19: An open-label, randomised, phase 2 trial. Lancet.

[18] Fricke-Galindo I., Falfán-Valencia R. (2021). Genetics Insight for COVID-19 Susceptibility and Severity: A Review. Front. Immunol..

[19] Carter-Timofte M.E., Jørgensen S.E., Freytag M.R., Thomsen M.M., Andersen N.-S.B., Al-Mousawi A., Hait A.S., Mogensen T.H. (2020). Deciphering the Role of Host Genetics in Susceptibility to Severe COVID-19. Front. Immunol..

[20] Lvovs D., Favorova O.O., Favorov A.V. (2012). A Polygenic Approach to the Study of Polygenic Diseases. Acta Naturae.

[21] The Severe COVID-19 GWAS Group (2020). Genome Association Study of Severe COVID-19 with Respiratory Failure. N. Engl. J. Med..

[22] Moher D., Liberati A., Tetzlaff J., Altman D.G., the PRISMA Group (2009). Preferred reporting items for systematic reviews and meta-analyses: The PRISMA statement. BMJ.

[23] Russell C.K., Gregory D.M. (2003). Evaluation of qualitative research studies. Évid. Based Nurs..

[24] Aromataris E., Fernandez R., Godfrey C.M., Holly C., Khalil H., Tungpunkom P. (2015). Summarizing systematic reviews. Int. J. Evid. Based Heal..

[25] Walsh D., Downe S. (2006). Appraising the quality of qualitative research. Midwifery.

[26] Pairo-Castineira E., Clohisey S., Klaric L., Bretherick A.D., Rawlik K., Pasko D., Walker S., Parkinson N., Fourman M.H., Russell C.D. (2021). Genetic mechanisms of critical illness in COVID-19. Nature.

[27] Horowitz J.E., Kosmicki J.A., Damask A., Sharma D., Roberts G.H.L., Justice A.E., Banerjee N., Coignet M.V., Yadav A., Leader J.B. (2022). Genome-wide analysis provides genetic evidence that ACE2 influences COVID-19 risk and yields risk scores associated with severe disease. Nat. Genet..

[28] Dieter C., Brondani L.d.A., Lemos N.E., Schaeffer A.F., Zanotto C., Ramos D.T., Girardi E., Pellenz F.M., Camargo J.L., Moresco K.S. (2023). Polymorphisms in *ACE1*, *TMPRSS2*, *IFIH1*, *IFNAR2*, and *TYK2* Genes Are Associated with Worse Clinical Outcomes in COVID-19. Genes.

[29] Degenhardt F., Ellinghaus D., Juzenas S., Lerga-Jaso J., Wendorff M., Maya-Miles D., Uellendahl-Werth F., ElAbd H., Arora J., Lenning O.B. (2022). Detailed stratified GWAS analysis for severe COVID-19 in four European populations. Hum. Mol. Genet..

[30] Liu D., Yang J., Feng B., Lu W., Zhao C., Li L. (2021). Mendelian randomization analysis identified genes pleiotropically associated with the risk and prognosis of COVID-19. J. Infect..

[31] Gaziano L., Giambartolomei C., Pereira A.C., Gaulton A., Posner D.C., Swanson S.A., Ho Y.-L., Iyengar S.K., Kosik N.M., Vujkovic M. (2021). Actionable druggable genome-wide Mendelian randomization identifies repurposing opportunities for COVID-19. Nat. Med..

[32] Ma Y., Huang Y., Zhao S., Yao Y., Zhang Y., Qu J., Wu N., Su J. (2021). Integrative genomics analysis reveals a 21q22.11 locus contributing risk to COVID-19. Hum. Mol. Genet..

[33] Fricke-Galindo I., Martínez-Morales A., Chávez-Galán L., Ocaña-Guzmán R., Buendía-Roldán I., Pérez-Rubio G., Hernández-Zenteno R.d.J., Verónica-Aguilar A., Alarcón-Dionet A., Aguilar-Duran H. (2022). IFNAR2 relevance in the clinical outcome of individuals with severe COVID-19. Front. Immunol..

[34] Nhung V.P., Ton N.D., Ngoc T.T.B., Thuong M.T.H., Hai N.T.T., Oanh K.T.P., Hien L.T.T., Thach P.N., Van Hai N., Ha N.H. (2022). Host Genetic Risk Factors Associated with COVID-19 Susceptibility and Severity in Vietnamese. Genes.

[35] Welzel T.M., Morgan T.R., Bonkovsky H.L., Naishadham D., Pfeiffer R.M., Wright E.C., Hutchinson A.A., Crenshaw A.T., Bashirova A., Carrington M. (2009). Variants in interferon-alpha pathway genes and response to pegylated interferon-Alpha2a plus ribavirin for treatment of chronic hepatitis C virus infection in the hepatitis C antiviral long-term treatment against cirrhosis trial. Hepatology.

[36] Smieszek S.P., Polymeropoulos V.M., Xiao C., Polymeropoulos C.M., Polymeropoulos M.H. (2021). Loss-of-function mutations in IFNAR2 in COVID-19 severe infection susceptibility. J. Glob. Antimicrob. Resist..

[37] Khanmohammadi S., Rezaei N., Khazaei M., Shirkani A. (2022). A Case of Autosomal Recessive Interferon Alpha/Beta Receptor Alpha Chain (IFNAR1) Deficiency with Severe COVID-19. J. Clin. Immunol..

[38] Zhang Q., Bastard P., Liu Z., Le Pen J., Moncada-Velez M., Chen J., Ogishi M., Sabli I.K.D., Hodeib S., Korol C. (2020). Inborn errors of type I IFN immunity in patients with life-threatening COVID-19. Science.

[39] Duncan C.J., Skouboe M.K., Howarth S., Hollensen A.K., Chen R., Børresen M.L., Thompson B.J., Spegarova J.S., Hatton C.F., Stæger F.F. (2022). Life-threatening viral disease in a novel form of autosomal recessive *IFNAR2* deficiency in the Arctic. J. Exp. Med..

[40] Jalkanen J., Khan S., Elima K., Huttunen T., Wang N., Hollmén M., Elo L.L., Jalkanen S. (2023). Polymorphism in interferon alpha/beta receptor contributes to glucocorticoid response and outcome of ARDS and COVID-19. Crit. Care.

[41] Raza R.Z., Abbasi S.W. (2022). An Evolutionary Insight Into the Heterogeneous Severity Pattern of the SARS-CoV-2 Infection. Front. Genet..

[42] Pahl M.C., Le Coz C., Su C., Sharma P., Thomas R.M., Pippin J.A., Cabrera E.C., Johnson M.E., Leonard M.E., Lu S. (2022). Implicating effector genes at COVID-19 GWAS loci using promoter-focused Capture-C in disease-relevant immune cell types. Genome Biol..

[43] Li P., Ke Y., Shen W., Shi S., Wang Y., Lin K., Guo X., Wang C., Zhang Y., Zhao Z. (2022). Targeted screening of genetic associations with COVID-19 susceptibility and severity. Front. Genet..

[44] Edahiro R., Shirai Y., Takeshima Y., Sakakibara S., Yamaguchi Y., Murakami T., Morita T., Kato Y., Liu Y.-C., Motooka D. (2023). Single-cell analyses and host genetics highlight the role of innate immune cells in COVID-19 severity. Nat. Genet..

[45] Akter S., Roy A.S., Tonmoy M.I.Q., Islam S. (2022). Deleterious single nucleotide polymorphisms (SNPs) of human IFNAR2 gene facilitate COVID-19 severity in patients: A comprehensive *in silico* approach. J. Biomol. Struct. Dyn..

[46] Duncan C.J.A., Mohamad S.M.B., Young D.F., Skelton A.J., Leahy T.R., Munday D.C., Butler K.M., Morfopoulou S., Brown J.R., Hubank M. (2015). Human IFNAR2 deficiency: Lessons for antiviral immunity. Sci. Transl. Med..

[47] Ma N., Zhang X., Yang L., Zhou J., Liu W., Gao X., Yu F., Zheng W., Ding S., Gao P. (2018). Role of Functional *IFNL4*, *IFNLR1*, *IFNA, IFNAR2* Polymorphisms in Hepatitis B virus-related liver disease in Han Chinese population. J. Viral Hepat..

[48] He X.-X., Chang Y., Jiang H.-J., Tang F., Meng F.-Y., Xie Q.-H., Li P.-Y., Song Y.-H., Lin J.-S., Zou R. Persistent Effect of IFNAR-1 Genetic Polymorphism on the Long-Term Pathogenesis of Chronic HBV Infection. www.liebertonline.com.

[49] Wada M., Marusawa H., Yamada R., Nasu A., Osaki Y., Kudo M., Nabeshima M., Fukuda Y., Chiba T., Matsuda F. (2009). Association of genetic polymorphisms with interferon-induced haematologic adverse effects in chronic hepatitis C patients. J. Viral Hepat..

[50] Hadjadj J., Yatim N., Barnabei L., Corneau A., Boussier J., Smith N., Péré H., Charbit B., Bondet V., Chenevier-Gobeaux C. (2020). Impaired Type I Interferon Activity and Inflammatory Responses in Severe COVID-19 Patients. Science.

[51] Davidson S., Maini M.K., Wack A., Jafarzadeh A., Nemati M., Saha B., Bansode Y.D., Jafarzadeh S. (2015). Disease-promoting effects of type i interferons in viral, bacterial, and coinfections. J. Interf. Cytokine Res..

[52] Prokunina-Olsson L., Alphonse N., Dickenson R.E., Durbin J.E., Glenn J.S., Hartmann R., Kotenko S.V., Lazear H.M., O’brien T.R., Odendall C. (2020). COVID-19 and emerging viral infections: The case for interferon lambda. J. Exp. Med..

[53] Hwang S.Y., Hertzog P.J., Holland K.A., Sumarsono S.H., Tymms M.J., Hamilton J.A., Whitty G., Bertoncello I., Kola I. (1995). A Null Mutation in the Gene Encoding a Type I Interferon Receptor Component Eliminates Antiproliferative and Antiviral Responses to Interferons a and f8 and Alters Macrophage Responses (Gene Targeting/Viral Infection/Macrophages/Signal Transduction). https://www.pnas.org.

[54] Langer J.A. Interferon at 50: New Molecules, New Potential, New (and Old) Questions Downloaded from. www.stke.org/cgi/content/full/2007/405/pe53http://stke.sciencemag.org/.

[55] Mantlo E., Bukreyeva N., Maruyama J., Paessler S., Huang C. (2020). Antiviral activities of type I interferons to SARS-CoV-2 infection. Antivir. Res..

[56] Lokugamage K.G., Hage A., de Vries M., Valero-Jimenez A.M., Schindewolf C., Dittmann M., Rajsbaum R., Menachery V.D. (2020). Type I Interferon Susceptibility Distinguishes SARS-CoV-2 from SARS-CoV. J. Virol..

[57] Busnadiego I., Fernbach S., Pohl M.O., Karakus U., Huber M., Trkola A., Stertz S., Hale B.G. (2020). Antiviral activity of type i, ii, and iii interferons counterbalances ace2 inducibility and restricts SARS-CoV-2. Mbio.

[58] Vanderheiden A., Ralfs P., Chirkova T., Upadhyay A.A., Zimmerman M.G., Bedoya S., Aoued H., Tharp G.M., Pellegrini K.L., Manfredi C. (2020). Type I and Type III Interferons Restrict SARS-CoV-2 Infection of Human Airway Epithelial Cultures. J. Virol..

[59] Zhang Q., Matuozzo D., Le Pen J., Lee D., Moens L., Asano T., Bohlen J., Liu Z., Moncada-Velez M., Kendir-Demirkol Y. (2022). Recessive inborn errors of type I IFN immunity in children with COVID-19 pneumonia. J. Exp. Med..

[60] Gilli F. (2010). Role of differential expression of interferon receptor isoforms on the response of multiple sclerosis patients to therapy with interferon beta. J. Interf. Cytokine Res..

[61] de Weerd N.A., Matthews A.Y., Pattie P.R., Bourke N.M., Lim S.S., Vivian J.P., Rossjohn J., Hertzog P.J. (2017). A hot spot on interferon α/β receptor subunit 1 (IFNAR1) underpins its interaction with interferon-β and dictates signaling. J. Biol. Chem..

[62] Zoellner N., Coesfeld N., De Vos F.H., Denter J., Xu H.C., Zimmer E., Knebel B., Al-Hasani H., Mossner S., Lang P.A. (2022). Synthetic mimetics assigned a major role to IFNAR2 in type I interferon signaling. Front. Microbiol..

